# Everolimus in acute kidney injury in a patient with breast cancer: a case report

**DOI:** 10.1186/1752-1947-8-386

**Published:** 2014-11-25

**Authors:** Francesca Donders, Dirk Kuypers, Pascal Wolter, Patrick Neven

**Affiliations:** 1 Department of Obstetrics and Gynecology - Gyn Oncol, University Hospitals Leuven/KU Leuven-University of Leuven, Herestraat 49, 3000 Leuven, Belgium; 2 Department of Nephrology, University Hospitals Leuven/KU Leuven-University of Leuven, Herestraat 49, 3000 Leuven, Belgium; 3 Department of General Medical Oncology, University Hospitals Leuven/KU Leuven-University of Leuven, Herestraat 49, 3000 Leuven, Belgium

**Keywords:** Acute renal injury, Breast cancer, Everolimus, Metastatic

## Abstract

**Introduction:**

Everolimus, a mammalian target of Rapamycin inhibitor, has recently been approved for the treatment of metastatic estrogen receptor-positive breast cancer, in combination with exemestane at a daily dose of 10mg. In the literature, few cases of acute kidney injury have been reported related to everolimus use, but none of them in a patient with breast cancer as we report here. Our case report of acute kidney injury demonstrates the potential nephrotoxic effects of everolimus therapy, necessitating close monitoring of renal function prior to, during and after discontinuation of the drug.

**Case presentation:**

We report the first published case of acute kidney injury shortly after initiation of exemestane and everolimus for metastatic breast cancer resistant to letrozole in a 69-year-old Caucasian woman, initially treated for a stage IIB estrogen receptor-positive breast cancer in 1997. Within 2 weeks of therapy, she developed grade 1 to 2 diarrhea, lower extremity edema, lethargy, and anorexia. After 4 weeks of therapy, her blood pressure was 85/59mmHg and she lost 4kg bodyweight. Her serum creatinine was 3.34mg/dL. Everolimus was stopped, and she was hospitalized for rehydration. Her serum creatinine levels peaked at 8.85mg/dL 8 days after treatment discontinuation, with a calculated creatinine clearance of 7mL/minute. Dialysis was not required. A month later, her serum creatinine levels slowly dropped to 2.26mg/dL but did not return to baseline. No re-challenge of everolimus was attempted.

**Conclusions:**

Extreme vigilance should be used when prescribing everolimus for metastatic breast cancer. Although the exact cause of acute kidney injury in our case is unknown, dehydration must be avoided and renal function closely monitored after initiating therapy. Spontaneous recovery after drug discontinuation is possible.

## Introduction

Everolimus, a mammalian target of rapamycin (mTOR) inhibitor, has recently been approved by the US Food and Drug Administration and the European Medicines Agency, in combination with exemestane, for the treatment of hormone receptor-positive, human epidermal growth factor receptor 2-negative advanced breast cancer in postmenopausal women without symptomatic visceral disease and after recurrence or progression following a non-steroidal aromatase inhibitor. Everolimus is given ata daily dose of 10mg continuously. Renal function impairment and a few cases of acute kidney injury (AKI) have been related to everolimus for non-breast cancer indications [1, 2]. We describe here the second reported case in the literature of AKI in a patient with breast cancer treated with everolimus. We discuss the pathophysiology and relevant literature to conclude that everolimus can cause a serious decline in renal function. Kidney function should be closely monitored prior to, during, and after discontinuing treatment.

## Case presentation

A 69-year-old Caucasian woman was treated for stage IIB estrogen receptor (ER)-positive left breast cancer in 1997. Besides two cerebrovascular accidents with full recovery, she had no significant comorbidities apart from chronic atrial fibrillation for which she received treatment with a vitamin K antagonist. In November 2010, she developed metastatic lesions in bone, lymph nodes, lung and liver. Over a 2-year period she consecutively received three lines of endocrine agents including tamoxifen for 6 months, letrozole for 11 months and abiraterone for 5 months. In December 2010 she underwent palliative radiotherapy for painful lytic lesions; she received intravenous zoledronic acid therapy monthly. Maintenance home therapy included simvastatin, bisoprolol, losartan, lormetazepam, and phenprocoumon. Exemestane 25mg plus everolimus 10mg were started in November 2012, 1 month after she switched from the vitamin K antagonist to low-molecular-weight heparin because of possible drug interactions with everolimus, since metabolization of phenprocoumon occurs through the CYP3A4 pathway.

**Figure 1:**
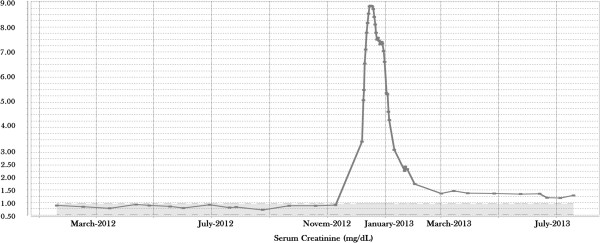
**The evolution serum creatinine levels (in mg/dL) in this patient with breast cancer, after starting everolimus treatment on November 9 and discontinuation on December 20 of 2012.**

At baseline, on October 19 of 2012, her renal function was normal based on her serum creatinine (0.87mg/dL; normal range, NR, 0.51 to 0.95) and estimated glomerular filtration rate (68mL/minute/1.73m^2^). On November 9 of 2012 the first cycle of everolimus was started. Her blood creatinine level was 0.90mg/dL. At the time of her first dose of everolimus an echocardiography was performed and showed no direct signs of left or right ventricular dysfunction. On December 7 she came for her week four visit to collect a second cycle of tablets, but a low blood pressure of 85/59mmHg was measured; she had lost 4kg bodyweight and an increase in serum creatinine to 3.34mg/dL was noted in the laboratory results. She reported that after 2 weeks of therapy she developed grade 1 to 2 diarrhea, leg edema, lethargy, and anorexia. She had another episode of watery diarrhea during the preceding week, but it had resolved by the time of her second dose. Everolimus was stopped and she was hospitalized and started on intravenous fluids because of signs of dehydration and possible secondary acute tubular necrosis (ATN). A new echocardiography showed no changes in cardiac function. In the following days, despite rehydration therapy with 1.5L/day of sodium chloride, her serum creatinine levels further increased. Serum creatinine levels peaked at 8.85mg/dL, with a calculated creatinine clearance of 7mL/minute, 8 days after treatment discontinuation. A renal ultrasound excluded post-renal causes of acute renal failure and showed normal sized kidneys. Her C-reactive protein levels were elevated to 240mg/dL (NR≤5) without leukocytosis or fever. There was no neutropenia. A chest X-ray showed bilateral minimal basal pulmonary effusions, but no infiltrates suspicious for infection were observed. Urine and hemocultures remained sterile. Urine microscopy showed rare granular casts and <10% of dysmorphic erythrocytes and few leukocytes. A 24-hour urine collection showed a proteinuria of 0.71g/24 hours (NR≤0.15); no renal biopsy was performed. On admission, immunological screening was negative and everolimus blood levels were 40ng/mL 4 hours post-dose. Dialysis was not required and 1 month after treatment discontinuation, her serum creatinine levels slowly dropped to 2.26mg/dL but never returned to baseline (Figure 1). No re-challenge of everolimus was attempted.

## Discussion

Two recent articles highlight nephrotoxicity associated with the use of mTOR inhibitors. Izzedine *et al.* (2013) reported the first four cases of AKI while Choueiri *et al.* (2013) considered AKI in all placebo-controlled trials to be the second most cause of fatal adverse events during early stages of therapy; however, in these studies, the patients did not have breast cancer [1, 2]. In the BOLERO-2 study, which showed a significant progression-free survival advantage of everolimus plus exemestane over exemestane alone, AKI has been reported in only one of the 482 patients with breast cancer [3].

First of all we need to take into account the cardiovascular comorbidity and her age in possibly overestimating renal functioning at baseline by only measuring serum creatinine. Most likely the AKI occurred following severe volume depletion, inducing ATN, which in turn required intact intracellular mTOR to enable tubular regeneration, in support of the autophagy theory [4]. A possible interaction of mTOR inhibitors with simvastatin, leading to a toxic dose of simvastatin and/or everolimus, through a common (cytochrome) metabolic pathway could have also played a role. Blood everolimus levels were 40ng/mL 4 hours after drug administration. This concentration falls within the expected range, in comparison to the results of the pharmacokinetic (PK) analyses of BOLERO-2, given that the median peak concentration 1 to 2 hours post-dose is 59.7 ±16.9 (standard deviation) ng/mL and the median trough concentration (Cmin) is 15.6 ±12.2ng/mL. Statin levels in our case were not measured. Co-administration of statins with everolimus could have induced rhabdomyolysis. Creatinine kinase levels, however, were only mildly raised (320U/L) and too low to be causal. Substrates of CYP3A4, such as HMG-CoA reductase inhibitors, are not expected to affect blood levels of everolimus. In BOLERO-2, 23% of everolimus-treated patients (versus 17% of patients treated with exemestane alone) received HMG-CoA reductase inhibitors as co-medications while AKI was reported in only one case in BOLERO-2 [3]. Exemestane itself is also metabolized by CYP3A4 and although everolimus, being a CYP3A4 inhibitor, increases exemestane levels, exemestane does not seem to affect everolimus blood levels [5]. However, PK analyses in BOLERO-2 showed that co-administration of exemestane had no effect on blood levels and overall exposure to everolimus. Co-administering everolimus with strong CYP3A4 inhibitors has been shown to increase Cmin and thus exposure to everolimus at maximum by 20% without having an effect on toxicity whatever grade [6]. It remains possible that intrarenal concentrations of everolimus or its pharmacodynamic (PD) effect are significantly affected by co-administration of exemestane despite acceptable blood concentrations [7].

There is now extensive experience with everolimus in several tumor types, as well as experience with other mTOR inhibitors. Much of this information comes from large databases from the manufacturer and several clinical trials. The safety profile of everolimus in the BOLERO-2 study is very similar to the profile reported in other clinical trials. Adverse events with everolimus are mostly mild to moderate in severity. With careful monitoring, early identification, and appropriate intervention – including dose interruption or reduction – they are manageable and generally reversible. The serious adverse event rate in BOLERO-2 was 23% versus 12% in the exemestane-alone arm [3].

It is unclear whether it is necessary to monitor everolimus blood levels during treatment, as there is no identified relationship between blood levels obtained after a 10mg everolimus dose and occurrence of specific toxicities. Following a 10mg oral dose, the drug exposure ranges are well characterized, as described above. Another question is whether a lower dose should be started. The efficacious dose used in phase 3 trials was 10mg. Initial PK and PD studies showed that 10mg produces more complete target inhibition than 5mg. One study has reported the use of a stepwise dose-escalation schedule of everolimus in combination with paclitaxel, starting with 2.5mg and escalating to 5mg (full combination dose) over 2 weeks [8]. Efficacy and safety studies utilizing this approach starting with 5mg everolimus and escalating to 10mg in combination with exemestane for ER-positive breast cancer are currently under consideration. Secondly, in cases when concomitant drugs, known to interfere with the metabolism of everolimus (CYP3A4), are initiated, monitoring of blood levels could prove to be advantageous.

Renal function seems to decrease with increasing low doses of everolimus (3mg versus 1.5mg) [9]. The adverse event profile among elderly patients in BOLERO-2 was generally comparable to that of younger patients [10]. However, deaths due to any cause within 28 days of the last everolimus dose occurred in 6% of patients ≥65 years of age compared to 2% in patients <65 years of age and adverse events leading to permanent treatment discontinuation occurred in 33% of patients ≥65 years of age compared to 17% in patients <65 years of age. Careful monitoring of older patients is therefore recommended.

## Conclusions

Despite an overall improved effect on kidney function following kidney transplantation and in specific types of renal diseases, a dose-dependent renal impairment has previously been reported with much lower doses of everolimus in transplant patients [9, 11]. Extreme vigilance should be used when prescribing everolimus for metastatic breast cancer. Although the exact cause of AKI in our case is unknown, dehydration should be avoided and renal function closely monitored prior to and soon after initiating everolimus 10mg.

## Consent

The patient is deceased. Written informed consent was obtained from the patient’s next of kin for publication of this case report and accompanying images. A copy of the written consent is available for review by the Editor-in-Chief of this journal.

## Authors’ information

PN – Prof. Dr in Obstetrics and Gynecology, Breast Oncology Specialist, Multidisciplinary Breast Centre University Hospitals Leuven (Belgium); involved in research and in national and international breast cancer studies.

FD – Medical student at University of Leuven (Belgium), Sub-Intern in Obstetrics and Gynecology, Multidisciplinary Breast Centre University Hospitals Leuven (Belgium); research on everolimus and breast cancer.

DK – Prof. Dr in Internal Medicine, subspecialty Nephrology at University Hospitals Leuven (Belgium).

PW – Prof. Dr in Internal Medicine, subspecialty General Medical Oncology at University Hospitals Leuven (Belgium).

## Abbreviations

AKI: Acute kidney injury
ATN: Acute tubular necrosis
Cmin: Median trough concentration
ER: Estrogen receptor
mTOR: Mammalian target of rapamycin
NR: Normal range
PD: Pharmacodynamic
PK: Pharmacokinetic.

## References

[1] Choueiri TK, Je Y, Sonpavde G, Richards CJ, Galsky MD, Nguyen PL, Schutz F, Heng DY, Kaymakcalan MD: **Incidence and risk of treatment-related mortality in cancer patients treated with the mammalian target of rapamycin inhibitors.** *Ann Oncol* 2013, **24:**2092–2097. 10.1093/annonc/mdt155 10.1093/annonc/mdt155 23658373

[2] Izzedine H, Escudier B, Rouvier P, Gueutin V, Varga A, Bahleda R, Soria JC: **Acute tubular necrosis associated with mTOR inhibitor therapy: a real entity biopsy-proven.** *Ann Oncol* 2013, **24:**2421–2425. 10.1093/annonc/mdt233 10.1093/annonc/mdt233 23798615

[3] Baselga J, Campone Campone M, Piccart M, Burris HA 3rd, Rugo HS, Sahmoud T, Noguchi S, Gnant M, Pritchard KI, Lebrun F, Beck JT, Ito Y, Yardley D, Deleu I, Perez A, Bachelot T, Vittori L, Xu Z, Mukhopadhyay P, Lebwohl D, Hortobagyi GN: **Everolimus in postmenopausal hormone-receptor-positive advanced breast cancer.** *N Engl J Med* 2012, **366:**520–529. 10.1056/NEJMoa1109653 10.1056/NEJMoa1109653 PMC5705195 22149876

[4] Nakagawa S, Nishihara K, Inui K, Masuda S: **Involvement of autophagy in the pharmacological effects of the mTOR inhibitor everolimus in acute kidney injury.** *Eur J Pharmacol* 2012, **696:**143–154. 10.1016/j.ejphar.2012.09.010 10.1016/j.ejphar.2012.09.010 23022334

[5] Kamdem LK, Flockhart DA, Desta Z: ***In vitro*****cytochrome P450-mediated metabolism of exemestane.** *Drug Metab Dispos* 2011, **39:**98–105. 10.1124/dmd.110.032276 10.1124/dmd.110.032276 PMC3014267 20876785

[6] Ravaud A, Urva SR, Grosch K, Cheung WK, Anak O, Sellami DB: **Relationship between everolimus exposure and safety and efficacy: Meta-analysis of clinical trials in oncology.** *Eur J Cancer* 2014, **50:**486–495. 10.1016/j.ejca.2013.11.022 10.1016/j.ejca.2013.11.022 24332451

[7] Podder H, Stepkowski SM, Napoli KL, Clark J, Verani RR, Chou TC, Kahan BD: **Pharmacokinetic interactions augment toxicities of sirolimus/cyclosporine combinations.** *J Am Soc Nephrol* 2001, **12:**1059–1071. 10.1681/ASN.V1251059 11316866

[8] Huober J, Fasching PA, Hanusch C, Rezai M, Eidtmann H, Kittel K, Hilfrich J, Schwedler K, Blohmer JU, Tesch H, Gerber B, Höβ C, Kümmel S, Mau C, Jackisch C, Khandan F, Costa SD, Krabisch P, Loibl S, Nekljudova V, Untch M, Minckwitz G: **Neoadjuvant chemotherapy with paclitaxel and everolimus in breast cancer patients with non-responsive tumours to epirubicin/cyclophosphamide (EC) +/- bevacizumab – results of the randomised GeparQuinto study (GBG 44).** *Eur J Cancer* 2013, **49:**2284–2293. 10.1016/j.ejca.2013.02.027 10.1016/j.ejca.2013.02.027 23541564

[9] Eisen HJ, Tuzcu EM, Dorent R, Kobashigawa J, Mancini D, Valantine-von Kaeppler HA, Starling RC, Sørensen K, Hummel M, Lind JM, Abeywickrama KH, Bernhardt P, RAD B253 Study Group: **Everolimus for the prevention of allograft rejection and vasculopathy in cardiac-transplant recipients.** *N Engl J Med* 2003, **349:**847–858. 10.1056/NEJMoa022171 10.1056/NEJMoa022171 12944570

[10] Pritchard KI, Burris HA 3rd, Ito Y, Rugo HS, Dakhil S, Hortobagyi GN, Campone M, Csöszi T, Baselga J, Puttawibul P, Piccart M, Heng D, Noguchi S, Srimuninnimit V, Bourgeois H, Gonzalez Martin A, Osborne K, Panneerselvam A, Taran T, Sahmoud T, Gnant M: **Safety and efficacy of everolimus with exemestane vs. exemestane alone in elderly patients with HER2-negative, hormone receptor-positive breast cancer in BOLERO-2.** *Clin Breast Cancer* 2013, **13:**421–432. 10.1016/j.clbc.2013.08.011 10.1016/j.clbc.2013.08.011 24267730

[11] Ponticelli C: **The pros and the cons of mTOR inhibitors in kidney transplantation.** *Expert Rev Clin Immunol* 2014, **10:**295–305. 10.1586/1744666X.2014.872562 10.1586/1744666X.2014.872562 24377908

